# Impact of diagnostic guidelines on the diagnosis of hypersensitivity pneumonitis

**DOI:** 10.3389/fmed.2023.1109525

**Published:** 2023-03-03

**Authors:** Yuh Chin Huang, Jessie P. Gu

**Affiliations:** Department of Medicine, Duke University Medical Center, Durham, NC, United States

**Keywords:** hypersensitivity pneumonitis, inciting agent, environmental exposure, pulmonary function test, guidelines and recommendations

## Abstract

**Introduction:**

Hypersensitivity pneumonitis (HP) is an immune-mediated interstitial lung disease from exposure to environmental antigens. Diagnosing HP could be challenging. The American College of Chest Physicians (CHEST) and American Thoracic Society/Japanese Respiratory Society/and Asociación Latinoamericana del Tórax (ATS/JRS/ALAT) have published diagnostic guidelines in 2021 and 2020 respectively. The CHEST guideline uses four grades of confidence: confident (>90%), provisional high (70–89%), provisional low (51–69%), and unlikely (≤50%). The ATS/JRS/ALAT guideline uses five grades of confidence: definite (>90%), high (80–89%), moderate (70–79%), low (51–69%) and not excluded (≤50%). In this study, we determined how these two guidelines could have affected the diagnosis of HP made before the guidelines.

**Methods:**

Two hundred and fifty-nine adult patients from a previous cohort with HP (ICD-9:495) made between Jan. 1, 2008, and Dec. 31, 2013, at Duke University Medical Center were included. We simplified the diagnostic confidence into three categories so we could compare the guidelines: high (≥90%), intermediate (51–89%), and low (≤50%).

**Results:**

There were 156 female and 103 male. Mean age was 58 (range: 20–90). 68.8% of the patients had restrictive defects (FVC < 80% pred) and 48.6% had lung biopsy. The CHEST guideline classified 33.6% of the patients into high, 59.5% into intermediate and 6.9% into low confidence categories. The ATS/JRS/ALAT guideline classified 29.7% of the patients into high, 21.2% into intermediate and 49.0% into low confidence categories (*p* < 0.0001 vs. CHEST). Cohen's kappa was 0.331. In patients with identifiable inciting agents (IAs) (*N* = 168), the CHEST guideline classified 32.1% of the patients into high, 64.3% into intermediate and 3.6% into low confidence categories. The ATS/JRS/ALAT guideline classified 29.2% of the patients into high, 20.8% into intermediate, and 50.0% into low confidence categories. Cohen's kappa was 0.314.

**Discussion:**

In our HP cohort with two-thirds of the patients with restrictive defects, we found the two guidelines had fair agreement in diagnosing HP with or without identifiable IAs. They agreed more when the diagnostic confidence was high. When the diagnostic confidence was lower, however, the ATS/JRS/ALAT guideline was more stringent. Clinicians should be aware of the differences between the two guidelines when evaluating patients suspicious of HP.

## Introduction

Diagnosing chronic hypersensitivity pneumonitis (HP) and distinguishing this disease from other inflammatory and fibrotic interstitial lung disease (ILD) can be challenging for the clinicians due to the heterogeneous clinical presentations, the difficulty in identifying inciting agents (IA) and variable radiologic and histopathologic patterns (1–6). Recently, the American College of Chest Physicians (CHEST) (7) and American Thoracic Society/Japanese Respiratory Society/and Asociación Latinoamericana del Tórax (ATS/JRS/ALAT) (8) have published diagnostic guidelines for HP in 2021 and 2020 respectively. Both guidelines include antigen identification, high-resolution computed tomography (HRCT) of the lungs, and histopathology in the algorithms to establish diagnostic confidence. The CHEST guideline is based on a four-level ontological framework: confident (>90% probability), provisional high confidence (70–89% probability), provisional low confidence (51–69% probability) and unlikely (≤50% probability). The ATS/JRS/ALAT guideline uses five grades of confidence: definite (>90% probability), high confidence (80–89% probability), moderate confidence (70–79% probability), low confidence (51–69% probability) and not excluded (≤50% probability) (Table 1).

**Table 1:** Diagnostic confidence categories in the CHEST and the ATS/JRS/ALAT guidelines (7, 8).

**CHEST**	**Probability**	**ATS/JRS/ALAT**	**Probability**
Confident	≥90%	Definite	≥90%
Provisional high	70–89%	High confidence	80–89%
Provisional low	51–69%	Moderate confidence	70–79%
Unlikely	≤50%	Low confidence	51–69%
		Not excluded	≤50%

In this study, we sought to determine how these diagnostic guidelines could affect the clinician diagnosis of HP. We applied the algorithms to a cohort of patients who had a diagnosis of HP made before the guidelines were available.

## Materials and methods

### Patient population

We included adult patients from a cohort of patients who had HP diagnosed by a pulmonologist or an allergist (ICD-9 = 495) between Jan. 1, 2008, and Dec. 31, 2013 at Duke University Medical Center previously published (9). We reviewed medical records to obtain demographics, pulmonary function tests and information about IA. We also reviewed CT scan reports and histopathology reports. If the CT reports were not available for review, primarily those that were done at outside hospitals, we placed the CT findings in the indeterminate category in the algorithms. The exposure information, CT and pathology were entered into the diagnostic algorithms in the guidelines to determine the diagnostic confidence. We did not include BAL lymphocytosis in the algorithm because few patients in our cohort had differential cell count in BAL due to the local practice pattern at the time. The Duke University Health System Institutional Review Board approved this study (IRB number Pro00052804).

### Diagnostic classification

Because the CHEST guideline had 4 categories and the ATS/JRS/ALAT guideline had five categories, in order to compare directly across the two guidelines, we simplified the grouping into high (≥90% probability), intermediate (51–89% probability), and low (≤50% probability) categories.

### Statistical analysis

We used the Fisher's exact test to compare between the two guidelines the percentage of patients in each category. We calculated Cohen's kappa to assess the agreement between the two guidelines (10). Statistical analyses were performed using Microsoft Excel Office 16.

## Results

Two hundred and fifty-nine patients from a previous cohort of patients with HP (excluding two pediatric patients) were included (9). There were 156 female and 103 male with a mean age of 58 years (range: 20–90 years). The pulmonary function test results in all patients and in patients with known IA are shown in Table 2. Note that 68.8% of the patients had FVC < 80% pred consistent with restriction. 83.4% had CT scan reports and 48.6% had lung biopsy reports.

**Table 2:** Pulmonary function test in patients with or without identifiable inciting agents (IAs). Data = mean (standard deviation).

**Parameter (% pred)**	**All patients (*N* = 225)**	**Patient with identifiable IAs (*N* = 153)**
FVC	67.3 (21.4)	68.8 (22.4)
FEV1	66.9 (21.4)	68.2 (22.3)
FEV1/FVC	78.4 (10.0)	78.7 (9.7)
TLC	70.6 (20.7)	71.7 (20.5)
RV	72.8 (35.6)	72.9 (36.0)
DLCO	59.6 (24.6)	59.6 (24.6)

In all patients, The CHEST guideline classified 87 patients (33.6%) into high, 154 (59.5%) into intermediate, and 18 (6.9%) into low confidence categories (Table 3). The ATS/JRS/ALAT guideline classified 77 patients (29.7%) into high, 55 (21.2%) into intermediate and 127 (49.0%) into low confidence categories (*p* < 0.0001 vs. CHEST) (Table 3). Cohen's kappa was 0.331.

**Table 3:** Comparison of diagnostic confidence based on the CHEST guideline with that based on the ATS/JRS/ALAT guideline in all patients and those with identifiable inciting agents (IAs).

**Simplified diagnostic categories**		**All patients (*N* = 259)**	**Patients with identifiable IAs (*N* = 166)**
**CHEST**	**ATS/JRS/ALAT**		
High	High	76 (29.3%)	47 (28.6%)
High	Intermediate	11 (4.2%)	6 (3.6%)
High	Low	0 (0.0%)	0 (0.0%)
Intermediate	High	0 (0.0%)	0 (0.0%)
Intermediate	Intermediate	41 (15.8%)	28 (16.7%)
Intermediate	Low	113 (43.6%)	79 (47.0%)
Low	High	0 (0.0%)	0 (0.0%)
Low	Intermediate	4 (1.5%)	1 (0.6%)
Low	Low	14 (5.4%)	5 (3.0%)

For this study, the categories were simplified to high (≥90% probability), intermediate (51–89% probability), and low (≤50% probability). Note that the intermediate category includes provisional high and low confidence categories in the CHEST guideline and high, moderate, and low confidence categories in the ATS/JRS/ALAT guidelines.

In patients with identifiable inciting agents (IA) (*N* = 168), the CHEST guideline classified 54 patients (32.1%) into high, 108 (64.3%) into intermediate, and 6 (3.6%) into low confidence categories (Table 3). The ATS/JRS/ALAT guideline classified 49 patients (29.2%) into high, 35 (20.8%) to intermediate and 84 (50.0%) into low confidence categories (Table 3). Cohen's kappa was similar (0.314). In patients with no identifiable IAs (*N* = 82), 43 patients (52.4%) had lung pathology that either confirmed or excluded the diagnosis of HP.

## Discussion

In our cohort who had a diagnosis of HP before the guidelines were available, both CHEST and the ATS/JRS/ALAT guidelines placed approximately 30% of the patients with prior HP diagnosis in the high (≥90% probability) diagnostic confidence category. The CHEST guideline tended to give higher diagnostic confidence than the ATS/JRS/ALAT guideline while the ATS/JRS/ALAT guideline placed more patients in the low (≤50% probability) category. The agreement between the two guidelines based on the k statistics was fair (10).

A recent study compared the two guidelines among a cohort of patients with HP at the National Institute of Respiratory Diseases in Mexico (11). In that study, 18% of the patients with pathology were placed in the >90% probability category based on the ATS/JRS/ALAT guideline while 65% of the patients were classified into that category based on the CHEST guideline. The study did not find a difference in the number of patients in the ≤50% category between the ATS/JRS/ALAT guideline and the CHEST guideline. The difference in results between our study and the study of Buendia-Roldan et al. may be due to the different populations included in the studies. Also note that in our patients who had no lung pathology (*N* = 129), 108 patients were in the “not excluded” category by the ATS/JRS/ALAT guideline. Both studies agreed that the ATS/JRS/ALAT guideline is more stringent because it emphasizes lung pathology.

Although the presence of identifiable IAs did not affect the agreement rate between the two guidelines in our study, it remains important to search for potential exposure. Identification of IAs followed by avoidance of the exposure is associated with less fibrotic HP (12–15). Even in patients with advanced fibrotic HP, antigen avoidance may slow progression from inflammation to fibrosis (16–19). A recent workshop summarized the exposure assessment tools that can help identify the inciting antigens with higher sensitivity and specificity and can help the clinicians apply the two HP diagnostic guidelines more effectively (20).

Our study had few patients with differential cell count in BAL due to the local practice pattern. In the CHEST guideline, BAL lymphocytosis could help move patients with typical or compatible CT findings with indeterminate or unidentified antigen exposure into the provisional high category, but not the confident category. In the ATS/JRS/ALAT guideline, BAL lymphocytosis helps move up a level of confidence if the inciting antigens are known. BAL lymphocytosis, however, could not increase the diagnostic probability to the high confidence category when the CT scan was not typical, and the inciting antigens were indeterminate or unknown.

In conclusion, about one-third of patients with previous diagnosis of HP were placed in the high confidence category by either guideline. The two guidelines had fair agreement in diagnosing HP. They agreed more when the diagnostic probability was high. When the diagnostic confidence was lower, however, the ATS/JRS/ALAT guideline was more stringent and using this guideline could result in more biopsy procedures, especially for cases in the intermediate confidence category. Clinicians should be aware of the differences between the two guidelines when using the guidelines to evaluate patients suspicious of HP. Future research is needed to determine if the two guidelines may lead to different clinical outcomes, including treatment strategies and adverse effects.

## Data availability statement

The raw data supporting the conclusions of this article will be made available by the authors, without undue reservation.

## Ethics statement

The studies involving human participants were reviewed and approved by Duke University Health System Institutional Review Board. Written informed consent for participation was not required for this study in accordance with the national legislation and the institutional requirements.

## Author contributions

YH developed the concept, study design, and revised the manuscript. JG analyzed the data and provided the first draft of the manuscript. All authors contributed to the article and approved the submitted version.

## References

[1] Zacharisen MC. Idiopathic interstitial pneumonia: are we missing hypersensitivity pneumonitis? Ann Allergy Asthma Immunol. (2002) 88:4–6. 10.1016/S1081-1206(10)63584-3 11814276

[2] Glazer CS. Chronic hypersensitivity pneumonitis: important considerations in the work-up of this fibrotic lung disease. Curr Opin Pulm Med. (2015) 21:171–7. 10.1097/MCP.0000000000000137 25584937

[3] Kouranos V Jacob J Nicholson A Renzoni E. Fibrotic hypersensitivity pneumonitis: key issues in diagnosis and management. J Clin Med. (2017) 6:62. 10.3390/jcm6060062 28617305 PMC5483872

[4] Ochi J Ohtani Y Takemura T Akashi T Tateishi T Miyazaki Y. Histological variability and consequences in chronic bird-related hypersensitivity pneumonitis. Respirology. (2017) 22:1350–6. 10.1111/resp.13070 28513923

[5] Costabel U Miyazaki Y Pardo A Koschel D Bonella F Spagnolo P. Hypersensitivity pneumonitis. Nat Rev Dis Primers. (2020) 6:65. 10.1038/s41572-020-0191-z 32764620

[6] Barnes H Troy L Lee CT Sperling A Strek M Glaspole I. Hypersensitivity pneumonitis: current concepts in pathogenesis, diagnosis, and treatment. Allergy. (2022) 77:442–53. 10.1111/all.15017 34293188

[7] Fernandez Perez ER Travis WD Lynch DA Brown KK Johannson KA Selman M. Diagnosis and evaluation of hypersensitivity pneumonitis: CHEST guideline and expert panel report. Chest. (2021) 160:e97–e156. 10.1016/j.chest.2021.03.066 33861992

[8] Raghu G Remy-Jardin M Ryerson CJ Myers JL Kreuter M Vasakova M. Diagnosis of hypersensitivity pneumonitis in adults. An official ATS/JRS/ALAT clinical practice guideline. Am J Respir Crit Care Med. (2020) 202:e36–69. 10.1164/rccm.202005-2032ST 32706311 PMC7397797

[9] Gu JP Tsai CL Wysham NG Huang YT. Chronic hypersensitivity pneumonitis in the southeastern United States: an assessment of how clinicians reached the diagnosis. BMC Pulm Med. (2020) 20:32. 10.1186/s12890-020-1072-7 32024493 PMC7003360

[10] Cohen J. A coefficient of agreement for nominal scales. Educ Psychol Meas. (1960) 20:37–46. 10.1177/001316446002000104

[11] Buendia-Roldan I Aguilar-Duran H Johannson KA Selman M. Comparing the performance of two recommended criteria for establishing a diagnosis for hypersensitivity pneumonitis. Am J Respir Crit Care Med. (2021) 204:865–8. 10.1164/rccm.202105-1091LE 34233145 PMC8528521

[12] Allen DH Williams GV Woolcock AJ. Bird breeders hypersensitivity pneumonitis - progress studies of lung-function after cessation of exposure to provoking antigen. Am Rev Resp Dis. (1976) 114:555–66. 970736 10.1164/arrd.1976.114.3.555

[13] Degracia J Morell F Bofill JM Curull V Orriols R. Time of exposure as a prognostic factor in avian hypersensitivity pneumonitis. Respir Med. (1989) 83:139–43. 10.1016/S0954-6111(89)80230-6 2602597

[14] Gimenez A Storrer K Kuranishi L Soares MR Ferreira RG Pereira CC. Change in FVC and survival in chronic fibrotic hypersensitivity pneumonitis. Thorax. (2018) 73:391–2. 10.1136/thoraxjnl-2017-210035 28883091

[15] Barnes H Lu J Glaspole I Collard HR Johannson KA. Exposures associations with clinical phenotypes in hypersensitivity pneumonitis: a scoping review. Respir Med. (2021) 184:106444. 10.1016/j.rmed.2021.106444 33991845

[16] Hanak V Kalra S Aksamit TR Hartman TE Tazelaar HD Ryu JH. Hot tub lung: presenting features and clinical course of 21 patients. Respir Med. (2006) 100:610–5. 10.1016/j.rmed.2005.08.005 16194601

[17] Selman M Pardo A King TEJr. Hypersensitivity pneumonitis: insights in diagnosis and pathobiology. Am J Respir Crit Care Med. (2012) 186:314–24. 10.1164/rccm.201203-0513CI 22679012

[18] Morell F Villar A Ojanguren I Munoz X Cruz MJ Sansano I. Hypersensitivity pneumonitis and (idiopathic) pulmonary fibrosis due to feather duvets and pillows. Arch Bronconeumol. (2021) 57:87–93. 10.1016/j.arbr.2019.12.024 32059824

[19] Petnak T Thongprayoon C Baqir M Ryu JH Moua T. Antigen identification and avoidance on outcomes in fibrotic hypersensitivity pneumonitis. Eur Respir J. (2022) 60. 10.1183/13993003.01336-2021 35236720

[20] Johannson KA Barnes H Bellanger AP Dalphin JC Fernandez Perez ER Flaherty KR. Exposure assessment tools for hypersensitivity pneumonitis. An official american thoracic society workshop report. Ann Am Thorac Soc. (2020) 17:1501–9. 10.1513/AnnalsATS.202008-942ST 33258669 PMC7706597

